# Local and Context-Attention Adaptive LCA-Net for Thyroid Nodule Segmentation in Ultrasound Images

**DOI:** 10.3390/s22165984

**Published:** 2022-08-10

**Authors:** Zhen Tao, Hua Dang, Yueting Shi, Weijiang Wang, Xiaohua Wang, Shiwei Ren

**Affiliations:** 1 School of Information and Electronics, Beijing Institute of Technology, 5 South Zhongguancun Street, Haidian District, Beijing 100081, China; 2 Yangtze Delta Region Academy of Beijing Institute of Technology, Jiaxing 314019, China; 3 Beijing Institute of Technology, Chongqing Center for Microelectronics and Microsystems, Chongqing 401332, China

**Keywords:** thyroid nodule segmentation, transformers, local details, ultrasound images, computer-aided diagnosis

## Abstract

The thyroid nodule segmentation of ultrasound images is a critical step for the early diagnosis of thyroid cancers in clinics. Due to the weak edge of ultrasound images and the complexity of thyroid tissue structure, it is still challenging to accurately segment the delicate contour of thyroid nodules. A local and context-attention adaptive network (LCA-Net) for thyroid nodule segmentation is proposed to address these shortcomings, which leverages both local feature information from convolution neural networks and global context information from transformers. Firstly, since most existing thyroid nodule segmentation models are skilled at local detail features and lose some context information, we propose a transformers-based context-attention module to capture more global associative information for the network and perceive the edge information of the nodule contour. Secondly, a backbone module with 7×1, 1×7 convolutions and the activation function Mish is designed, which enlarges the receptive field and extracts more feature details. Furthermore, a nodule adaptive convolution (NAC) module is introduced to adaptively deal with thyroid nodules of different sizes and positions, thereby improving the generalization performance of the model. Simultaneously, an optimized loss function is proposed to solve the pixels class imbalance problem in segmentation. The proposed LCA-Net, validated on the public TN-SCUI2020 and TN3K datasets, achieves Dice scores of 90.26% and 82.08% and PA scores of 98.87% and 96.97%, respectively, which outperforms other state-of-the-art thyroid nodule segmentation models. This paper demonstrates the superiority of the proposed LCA-Net for thyroid nodule segmentation, which possesses strong generalization performance and promising segmentation accuracy. Consequently, the proposed model has wide application prospects for thyroid nodule diagnosis in clinics.

## 1. Introduction

The thyroid gland is honored as the ‘life gland’ of human beings, which is an essential endocrine organ in the body [1]. The thyroid regulates the body’s metabolism, growth and development by secreting thyroid hormones. Thyroid nodules are irregular lumps due to lesions in the thyroid gland, which are considered to be the main clinical manifestations of thyroid abnormalities [1,2,3]. Consequently, the diagnosis of thyroid lesions through thyroid nodules has become the most common diagnostic method. For the evaluation and diagnosis of thyroid nodules [3], ultrasound technology has become the optimal choice to provide nodules’ information owing to its non-invasive, practical and painless characteristics [4,5]. The thyroid nodule segmentation of ultrasound images is an indispensable step for the analysis of nodules’ characteristics and a prerequisite for accurate diagnosis [6]. Generally, radiologists need to carefully read ultrasound images and manually draw the edge contour of the nodules. However, the manual segmentation of a nodule contour is time-consuming and tedious. Meanwhile, on account of the intrinsic characteristics of ultrasound images, such as weak edges, heterogeneity [5] and low contrast [2], it is challenging to segment the delicate contours of thyroid nodules from ultrasound images. In order to mitigate these issues, several computer-aided diagnosis (CAD) systems are employed to precisely and automatically segment nodules to assist radiologists.

Before the rise of deep learning, the thyroid nodule segmentation of ultrasound images relied on conventional image processing technologies, which could be roughly divided into three categories [2]: contour and shape-based methods, region-based methods, and traditional machine learning methods [5,6]. Du et al. [7] proposed a novel DRLSE algorithm based on the contour and shape to segment thyroid nodules in ultrasound images. This method overcame a certain amount of ultrasonic image noise and nodule echogenicity and prevented the leakage of some boundaries of nodules. However, the DRLSE algorithm performed poorly in the segmentation of weak edges and was sensitive to the locations of manually initialized contours [7]. Zhao et al. [8] proposed a normalized cut method based on regions which could segment the thyroid nodules and the trachea areas in ultrasound images. The algorithm reduced the number of parameters, but the fineness of segmentation still needed to be improved. Keramidas et al. [9] utilized a fuzzy grey-level histogram for feature extraction and an SVM algorithm for pixels classification in order to segment the thyroid nodules. Nevertheless, this algorithm was sensitive to the selected features, and the segmentation accuracy remained to be improved. In summary, these conventional thyroid nodule segmentation methods cannot overcome the characteristics of ultrasound and thyroid tissue well and the segmentation accuracy still needs improvement.

With the remarkable development of deep learning, it has been applied to medical image diagnosis and provides auxiliary diagnosis advice in clinics [10]. In the field of thyroid noduls segmentation, models based on deep learning have significantly outperformed traditional algorithms [1,2,5]. Based on supervised learning, deep learning surmounts the inherent characteristics of ultrasound images and could distinguish the contour boundary between nodules and other thyroid tissues. In 2017, Ma et al. [11] first applied deep convolutional networks to the thyroid nodule segmentation of ultrasound images. This overcame the shortcomings of conventional methods such as manually extracting nodule features and improved the efficiency of automatic segmentation. However, this method lost plenty of feature details, leading to less-than-ideal segmentation results. In 2020, Yang et al. [5] adopted a modified UNet for the initial segmentation and then a level set algorithm based on the image feature direction to refine the segmentation performance. However, due to the inherent properties of convolution, such as gathering information only from neighborhood pixels [12], this network could not explicitly capture global dependency [13]. In 2021, Gong et al. [6] proposed a thyroid region prior guided feature enhancement model for thyroid nodule segmentation. This model could segment the thyroid gland and its internal nodules simultaneously, making it easier for radiologists to compare the tissues around the nodules for subsequent diagnosis. However, this network pays too much attention to global information but ignores the local feature details. Recently, Zhang et al. [3] reported segmenting thyroid nodules with a cascade UNet framework. The network utilized the first UNet to roughly locate the nodule position and employed the second UNet to perform fine segmentation to obtain the final result. Nevertheless, it is not robust to thyroid nodules of diverse sizes and locations. Given the original convolution networks capturing multi-scale features [12] in the limited scaling range by employing the 3×3 convolution and pooling operations [14,15] and lacking the ability to explicitly capture global dependency [13], it is still challenging to achieve high accuracy in the segmentation of thyroid nodules. It can be seen from the above that due to the various and complex shapes of thyroid nodules in the actual clinics, the existing thyroid nodule segmentation networks could not satisfactorily segment the contours of the nodules.

In this paper, a local and context-attention adaptive network (LCA-Net) is proposed to further overcome the shortcomings of the current thyroid nodule segmentation methods. This model could more accurately and automatically depict the contour of nodules and even better assist radiologists in the subsequent diagnosis of clinics. Considering the characteristic of convolutional neural networks focusing on local details and the merits of transformers in capturing global context information, this paper mainly employs CNNs and transformers to design the whole novel end-to-end network structure. Specifically, the backbone modules based on CNNs and the context-attention modules based on transformers designed in this paper are utilized to extract local feature details and global associative information. Simultaneously, a nodules adaptive convolutions (NAC) module is embedded into the encoder of the network, which assists the network to have promising adaptability for various sizes and positions of nodules. Furthermore, an optimized loss function is proposed to solve the pixels class imbalance problem in segmentation. Experiments are performed on the public TN-SCUI2020 dataset and TN3K dataset to evaluate our method, and the results are superior to the current thyroid nodule segmentation models.

The main contributions of our algorithm are five-fold:

- A novel local and context-attention adaptive network (LCA-Net) is proposed for thyroid nodule segmentation in ultrasound images. Compared with the existing state-of-the-art methods of thyroid nodule segmentation, the LCA-Net shows promising segmentation accuracy and strong generalization performance.
- A context-attention module based on transformers is proposed to gain global associative information to the model.
- A backbone module with 7×1, 1×7 convolutions and activation function Mish is designed to more finely extract the local feature details of nodules.
- The nodule adaptive convolutions (NAC) module is embedded into the encoder of the LCA-Net to enhance the robustness of the network to various sizes and positions of nodules.
- A novel loss function is proposed to solve the pixels’ class imbalance problem for thyroid nodule segmentation.

The rest of the paper is organized as follows. In Section 2, we provide the detailed description of our proposed method, introducing the overall architecture, each sub-module design and a novel loss function. In Section 3, the performance and robustness of the proposed method are evaluated over the two public thyroid nodule datasets using the standard performance metrics and the results are compared with the previous state-of-the-art works. Finally, Section 4 concludes the paper and suggests topics for future work.

## 2. Materials and Methods

This paper aimed to establish a high-accuracy and strong generalization thyroid nodule segmentation model to assist radiologists in clinical diagnosis. In this section, we propose a novel thyroid nodule segmentation scheme. Firstly, we introduce the proposed LCA-Net architecture which includes backbone modules, context-attention modules and a nodule adaptive convolution module. Afterward, we present the details of the context-attention module, the backbone module, the nodule adaptive convolution module and a novel loss function, respectively.

### 2.1. LCA-Net Architecture

**Figure 1:**
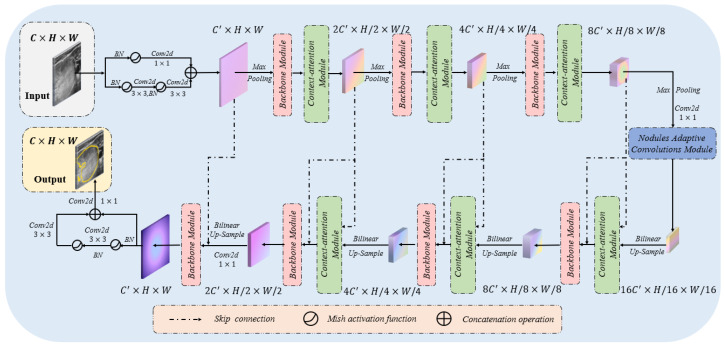
Illustration of the proposed local and context-attention adaptive network (LCA-Net) for the thyroid nodule segmentation of ultrasound images. The LCA-Net has the structure of an encoder and decoder. It mainly contains the backbone modules, context-attention modules and a nodule adaptive convolutions module, which could extract local details and global context information. See the text for more details.

Currently, existing thyroid nodule segmentation models in the literature are often not comprehensive enough in design, which ignores the global information capture ability or the generalization performance of the practical clinical application. Considering the weak edge and low contrast of thyroid nodules in ultrasound images, it is easy to misdiagnose using convolution neural networks to extract the local features of thyroid nodules. Hence, we designed a novel thyroid nodule segmentation model called LCA-Net from the perspective of taking global context, local details and generalization performance into account. Figure 1 shows the specific structure of the LCA-Net. This network based on the encoder–decoder framework is mainly composed of backbone modules and context-attention modules. Since the convolution neural networks are experts in extracting local detail features, our backbone module primarily consists of two consecutive 7×1 and 1×7 large convolution kernels, which are responsible for extracting more detailed edge features of nodules. However, the convolution kernel has the inherent characteristics of excessively paying attention to the detailed features of nodules and the middle part of feature maps. It is easy to lose some significant features only by using convolution neural networks to segment the contour of nodules. In actual clinical ultrasound images, the contour of the thyroid nodule is generally located at the edge of the region of interest [15], and convolution neural networks could not be fully considered. In view of the ability of Transformers to capture high-resolution context information in image segmentation, we designed a context-attention module based on transformers. Consequently, this module was integrated into the network, making it feasible for the LCA-Net to precisely segment the edge contours. Instead of naively integrating the Context-attention modules on top of the feature maps from the CNN backbone, we embedded the context-attention modules into each level of the encoder and decoder to collect long-range dependency from multiple scales. Moreover, the skip connection enables the context-attention module to capture more global features through the fusion of low-level features and high-level features. This framework enables each network encoding and decoding to give the extraction of local features and the context association information of ultrasound images into consideration to more finely perceive the contour information of thyroid nodules. It is worth noting that the LCA-Net employs the convolution kernels of 3×3 for the original image resolution because the input and output ports of the network pay more attention to the detailed textures. Therefore, simply embedding the backbone module or the context-attention module will affect the performance of the whole model and increase additional computation. Finally, we embedded the nodule adaptive convolution (NAC) module in the last part of the network encoder, which enables the network to adaptively deal with the thyroid nodules of various sizes and positions without additional computation [16,17]. The integration of this module also improves the generalization performance of the whole model in practical clinical application.

### 2.2. Context-Attention Module

**Figure 2:**
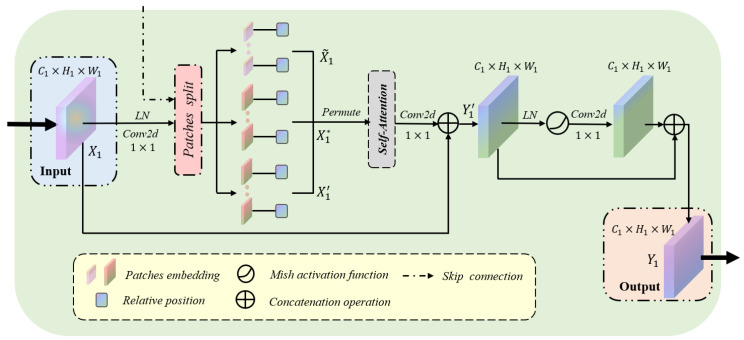
The specific structure of the context-attention module, where LN is the layer normalization operator, and $C_{1}\times H_{1}\times W_{1}$ represents the map shape (channel, height and width).

**Figure 3:**
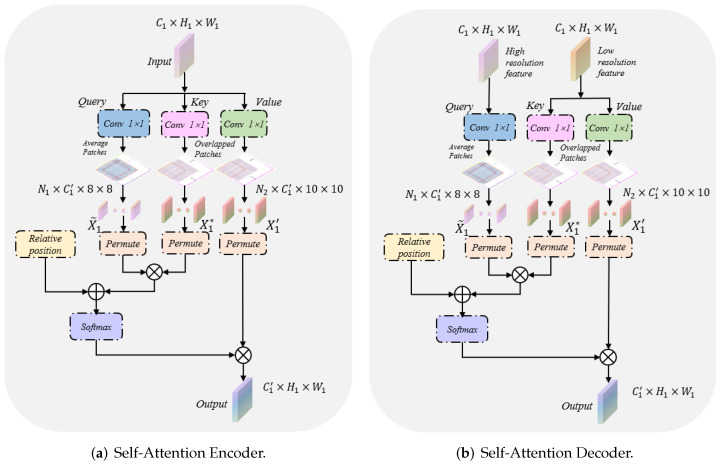
The proposed novel self-attention, where $N_{1}$, $N_{2}$ are the patch number, ⊗ is matrix multiplication and ⊕ denotes concatenation operation. (**a**,**b**) denote the encoder and decoder of self-attention, respectively. They share similar concepts. Significantly, the decoder of the self-attention takes high-resolution features from the encoder for query, and low-resolution features from the decoder for key and value.

Figure 1 highlights the locations of the context-attention modules. It is worth noting that we apply the context-attention modules to each level of the encoder and decoder to capture global association information from multiscale feature maps. In clinical practice, the edge contour of the thyroid nodule is often distributed at the boundary of ultrasound images. In this case, barely utilizing convolution neural networks cannot finely segment the nodule contour. In view of the outstanding performance of transformers in capturing the global pixel of images, we established a context-attention module based on transformers. The specific structure of the context-attention module is shown in Figure 2. However, we cannot directly capture the global information of the nodule feature map with the classical vision transformers [14,15]. Due to a large number of pixels in the ultrasound image, employing the classical vision transformer easily leads to problems such as excessive network calculation, over-fitting and challenging training. In order to alleviate the above problems and make the model aware of the global context, we preprocessed the nodule feature map before inputting the transformer. As seen in Figure 3a,b, firstly, three 1×1 convolution kernels are exploited to project input to query, key and value embeddings. Hence, the dimensions of query, key and value input into the module are: $Q,K,V\in R^{C_{1}^{{}^{\prime }}\times H_{1}\times W_{1}}$, where $C_{1}^{{}^{\prime }}$ denotes the dimension of embedding in self-attention mechanism, $H_{1}$, $W_{1}$ are the spatial height and width. Afterward, we divide the query, key and value embeddings into a fixed number of small patches instead of simple pixels. Specifically, the window size of the query patch is set to 8×8, while the size of the key-value patch is set to 10×10 and crossed. This leads to the feature size of the query of $Q\in R^{N_{1}\times C_{1}^{{}^{\prime }}\times 8\times 8}$, where the total number patches of the query is $N_{1}=\frac{H_{1}}{8}\times \frac{W_{1}}{8}$. Similarly, the feature size of the key-value is $K,V\in R^{N_{2}\times C_{1}^{{}^{\prime }}\times 10\times 10}$, and the number patches of the key-value is $N_{2}=\left(\frac{H_{1}-10}{s}+1\right)\times \left(\frac{W_{1}-10}{s}+1\right)$, where *s* is the step size of moving the key-value patches. Notably, the query is different from the key-value patch sizes, and the key-value patch is overlapped, which is larger than the query patch. Extensive experiments show that keeping larger and overlapping key-value patches leads to significant performance gains due to the small information exchange between two neighborhood windows. This design aims to save the amount of computation and maximize the transformer’s ability to capture contextual information as much as possible. Additionally, the function permute is used to transform the dimensions of *Q*, *K* and *V* to facilitate subsequent matrix operations. At this point, the dimensions of $\hat{Q}$, $\hat{K}$ and $\hat{V}$ after transformation are: $\hat{Q}\in R^{C_{1}^{{}^{\prime }}\times M_{1}}$, $\hat{K},\hat{V}\in R^{C_{1}^{{}^{\prime }}\times M_{2}}$, where $M_{1}=N_{1}\times 8\times 8$ and $M_{2}=N_{2}\times 10\times 10$. Simultaneously, the input of the transformer as the decoder is different from that of the encoder. In decoders, the query input comes from the skip connections of the encoder, while the key-value utilizes the output of the previous module as the input. Through the fusion of low-level features and high-level features, the context-attention module retains more global context information. Furthermore, considering the fact that ultrasound images are highly structured, we also introduce relative position index [15,18]. By recording the relative position between patches, the performance of the module is further promoted. Therefore, the proposed self-attention is defined as:

(1) $$Attention\left(\hat{Q},\hat{K},\hat{V}\right)=softmax\left(\frac{\hat{Q}⊗{\hat{K}}^{T}+\varphi }{\sqrt{C_{1}^{{}^{\prime }}}}\right)⊗\hat{V},$$

where $\varphi \in R^{M_{1}\times M_{2}}$ denotes relative position bias and ⊗ denotes matrix multiplication.

The proposed self-attention takes into account the information and position of all pixels in the feature map, so it can extract more global context features. We define the input feature map of the context-attention module as $X_{1}\in R^{C_{1}\times H_{1}\times W_{1}}$, where $C_{1}$, $H_{1}$, $W_{1}$ represent the channel number, the height and the width of the feature map. First, the channel number of $X_{1}$ is changed by 1×1 convolution operation, and $X_{1}$ is, respectively, split into a sequence of patches and transformed the dimensions: $\tilde{X_{1}}=\left[\tilde{x_{1}},...,\tilde{x_{N_{1}}}\right]\in R^{C_{1}^{{}^{\prime }}\times \left(N_{1}\times 8\times 8\right)}$ for $\hat{Q}$; $X_{1}^{*}=\left[x_{1}^{*},...,x_{N_{2}}^{*}\right]\in R^{C_{1}^{{}^{\prime }}\times \left(N_{2}\times 10\times 10\right)}$ for $\hat{K}$; and $X_{1}^{{}^{\prime }}=\left[x_{1}^{{}^{\prime }},...,x_{N_{2}}^{{}^{\prime }}\right]\in R^{C_{1}^{{}^{\prime }}\times \left(N_{2}\times 10\times 10\right)}$ for $\hat{V}$, where $N_{1}$, $N_{2}$ are the number of patches. The specific calculation process is shown in the following formula:

(2) $$\tilde{X_{1}},X_{1}^{*},X_{1}^{{}^{\prime }}=\Phi_{qkv}\left[f_{ps}\left(g\left(LN\left(X_{1}\right)\right)\right)\right].$$

We denote (·) as the dot-product operation, where LN(·) denotes the layer normalization operator, g(·) is the 1×1 convolution layer, $f_{ps}\left(\cdot \right)$ is the patches split operation, and $\Phi_{qkv}\left(\cdot \right)$ is the permute function. Subsequently, we input $\tilde{X_{1}},X_{1}^{*},X_{1}^{{}^{\prime }}$ into the self-attention to capture more global context information, and the specific process is summarized as follows:

(3) $$Y_{1}^{{}^{\prime }}=g\left[Attention\left(\tilde{X_{1}},X_{1}^{*},X_{1}^{{}^{\prime }}\right)\right]⊕X_{1}\in R^{C_{1}\times H_{1}\times W_{1}}.$$

Furthermore, the feature map passes through the rest of the module, and the calculation process can be defined as:

(4) $$Y_{1}=g\left[LN\left(Y_{1}^{{}^{\prime }}\right)\right]⊕Y_{1}^{{}^{\prime }}\in R^{C_{1}\times H_{1}\times W_{1}},$$

where ⊕ denotes the concatenation operation and g(·) represents the 1×1 convolution layer.

### 2.3. Backbone Module

**Figure 4:**
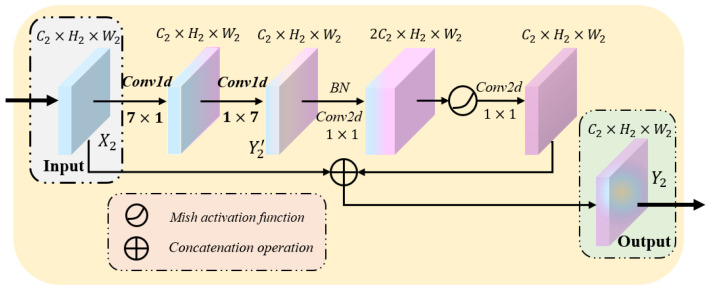
The specific structure of the backbone module, where BN denotes the batch normalization operator.

As shown in Figure 1, similarly to the locations of context-attention modules, we embed the backbone module to each level of the encoder and decoder to extract local feature details from multiple scales. The critical technology of thyroid nodule segmentation depends on extracting the detailed features of nodule contour. Due to the calculation principle of the sliding window, the convolution neural networks pay more attention to the information in the sliding window, that is, the local details. Therefore, we establish the backbone module based on CNNs. The specific structure of the backbone module is shown in Figure 4.

In general, because the smaller convolution kernel has merits such as better nonlinear expression and fewer parameters, researchers use it to build deep neural networks. Nevertheless, the smaller convolution kernel will also lead to the incomplete extraction of local information, resulting in the loss of thyroid nodule contour information. Simultaneously, the application of pooling layers further leads to the reduction in the image resolution and the loss of spatial feature information [17,19]. To alleviate these problems, we adopt a large convolution kernel with a size set of 7. This design not only enlarges the receptive field of the network and extracts more local detailed information but also offsets the loss of feature information as much as possible. Considering the shortcomings of the large convolution kernel, such as too large parameters and easy over-fitting, we employ two consecutive 7×1 and 1×7 convolution layers. Extensive experiments show that the effect of extracting nodule features from a 7×7 large convolution kernel is essentially the same as that of two consecutive convolutions with the size of 7. This design makes the network end up with more disentangled parameters and therefore with faster training [20]. At this point, the computational parameters of the module are reduced by approximately 72%. Consequently, the convolution layers of the module have better local feature extraction performance and fewer parameters.

As is known to all, the activation function is nonlinear, which improves the expression ability of the neural network to the model. Due to its fast convergence speed, no gradient saturation and gradient disappearance, the ReLU function has become the mainstream neural network activation function. Meanwhile, this function also has shortcomings such as neuron necrosis and not being smooth enough. Generally speaking, the smoother activation function can better transfer information and improve the accuracy and generalization ability of the model. Considering the fact that the loss of information should be avoided as much as possible when extracting the detailed features of nodules, an advanced activation function Mish [21] is introduced in this paper:

(5) $$y=x\cdot \tanh(ln\left(1+e^{x}\right))$$

**Figure 5:**
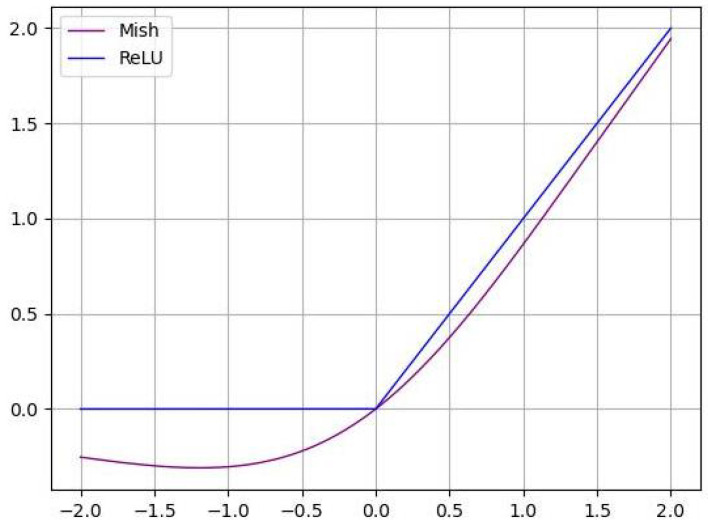
The activation function diagram of Mish and ReLU.

As seen in Figure 5, compared with the widely adopted activation function ReLU, the Mish function is smoother, so it shows more significant superiority in transmitting information and anti-interference.

The remaining two 1×1 convolution kernels in the backbone module are mainly used to transform feature map channels. We denote the input and output of the module as $X_{2},Y_{2}\in R^{C_{2}\times H_{2}\times W_{2}}$, where $C_{2}$, $H_{2}$ and $W_{2}$ represents the channel number for feature map, the length and width of the feature map. In the backbone module, the local detail features of the feature map are first extracted by two large convolution kernels as shown in Formula (5):

(6) $$Y_{2}^{{}^{\prime }}=f_{2}\left[f_{1}\left(X_{2}\right)\right]\in R^{C_{2}\times H_{2}\times W_{2}},$$

where $f_{1}\left(\cdot \right)$, $f_{2}\left(\cdot \right)$, respectively, represent two consecutive 7×1 and 1×7 convolution layers. Afterward, we utilize two 1×1 convolution kernels to change the number of feature map channels. The specific calculation process is shown in the following formula:

(7) $$Y_{2}=g\left[\sigma \left(g\left(BN\left(Y_{2}^{{}^{\prime }}\right)\right)\right)\right]⊕X_{2},$$

where g(·) represents the 1×1 convolution layer, BN(·) is the batch normalization operator, σ(·) denotes the activation function Mish and ⊕ is concatenation operation.

### 2.4. Nodule Adaptive Convolution Module

**Figure 6:**
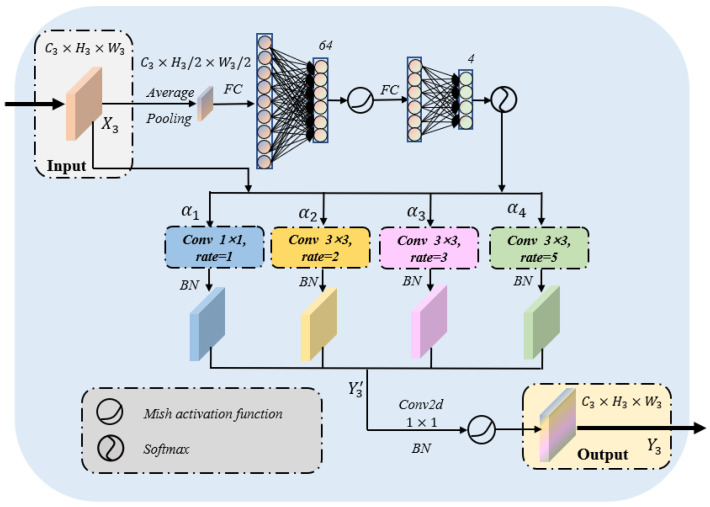
The specific structure of nodule adaptive convolution module. FC denotes the fully connected layer, and $\alpha_{1}$, $\alpha_{2}$, $\alpha_{3}$ and $\alpha_{4}$ represent the adaptive ratios of each convolution when dealing with different nodules.

In clinical practice, the size and location of thyroid nodules are various. Hence, it is difficult to merely match the variable nodules with the convolution kernel of fixed size [16]. The large convolution kernel could often extract the features of large or medium position objects, while the small convolution kernel is better for small or remote position targets. Therefore, it is of great significance to flexibly adjust the appropriate convolution according to the size of the nodule. In order to better adapt to the practical clinical application, we designed a nodule adaptive convolution (NAC) module to match various thyroid nodules. The specific structure of the module is shown in Figure 6. The main body of the module is the input preprocessing and aggregation of multiple convolution kernels of different sizes. The preprocessing part dynamically adjusts the weights of each convolution kernel according to the input, thereby generating adaptive dynamic convolutions. Simultaneously, dilated convolution is widely applied for neural network optimization because it enlarges the receptive field without adding additional parameters. As illustrated in Figure 6, we introduced four different ratios of dilated convolutions [17,19], from 1 to 2, 3 and 5, then the receptive field of each branch is 1, 5, 7 and 11. After four multi-scale feature maps are achieved, they will be spliced. This design could deal with nodules of different sizes and locations in actual clinical practice and improve the generalization ability of the model.

Let us denote the input and output of the module as $X_{3},Y_{3}\in R^{C_{3}\times H_{3}\times W_{3}}$, where $C_{3}$ is the number of feature channels, $H_{3}$ and $W_{3}$ are the length and width of the feature map. We define the calculation process of the NAC module by aggregating multiple dilated convolutions with different weights as follows:

(8) $$Y_{3}^{{}^{\prime }}=\sum_{k=1}^{K}\alpha_{k}\left(X_{3}\right)\cdot \sigma \left[BN\left(f_{k}\left(X_{3}\right)\right)\right],$$

(9) $$Y_{3}=\sigma \left[BN\left(g\left(Y_{3}^{{}^{\prime }}\right)\right)\right],$$

(10) $$0\leq \alpha_{k}\left(X_{3}\right)\leq 1,\sum_{k=1}^{K}\alpha_{k}\left(X_{3}\right)=1,$$

where $f_{k}\left(\cdot \right)$, $\alpha_{k}$ represent each dilated convolution and its assigned weight, respectively, σ(·) is the Mish activation function, BN(·) is the Batch normalization operator and g(·) denotes a 1×1 convolution layer which is used to adjust the number of channels $C_{3}$.

### 2.5. Loss Function for Pixels Class Imbalance

In actual ultrasound images, the thyroid nodule occupies only a small part of the image, while the rest are mostly background. This can easily cause pixel class imbalance, which affects the segmentation performance of the model. Therefore, if the conventional dice loss or cross-entropy loss is directly used to optimize the network parameters, the prediction segmentation maps in the test stage are not ideal. Motivated by focal loss structure [22], an optimized loss function is devised to overcome this problem. First, we introduce a weight factor ${\left(1-p_{x,y,c}\right)}^{2}$ into the cross-entropy loss, where $p_{x,y,c}$ represents the prediction probability of the real value at pixel (x,y), and *c* is the pixel class. Hence, the prediction probability of misclassified pixels is $\left(1-p_{x,y,c}\right)$. At this point, the value of the weighed factor is large, which is equivalent to increasing the weight of misclassified pixels in the loss function. Nevertheless, with the progress of the training process, if the model forever pays attention to the misclassified pixels, it will affect the accuracy of pixel classification. In order to further develop the performance of the loss function, we add $p_{x,y,c}^{2}$ as the fine-tuning of the function. As a consequence, we redefine the loss function $L_{LCA}$ as follows:

(11) $$L_{LCA}=-\frac{1}{HW}\sum_{x=0}^{H-1}\sum_{y=0}^{W-1}\sum_{c=0}^{1}R_{x,y,c}log\left(\frac{p_{x,y,c}^{{\left(1-p_{x,y,c}\right)}^{2}}+p_{x,y,c}^{2}}{2}\right),$$

where H,W are the height and width of map, *c* denotes the pixel class and $R_{x,y,c}$ represents the real value of pixels.

## 3. Experiments and Results

### 3.1. Datasets

In this paper, in order to realize the function of the LCA-Net, we needed to utilize the suitable thyroid nodule datasets of ultrasound images to train and test the model. The TN-SCUI2020 [2,23] and TN3k [5] datasets come from different institutions with different data sources and dataset sizes. Therefore, using these two datasets to verify the model performance can also reflect the generalization ability of the model.

#### 3.1.1. TN-SCUI2020

The TN-SCUI2020 dataset comes from the challenge of Thyroid Nodule Segmentation and Classification in Ultrasound Images, which provides a public 2D dataset of a thyroid nodules with approximately 7288 ultrasound images. These images were collected from different ages, genders and in different sites using various ultrasound machines such as Mindray DC-8, Philips-cx50 and TOSHIBA Aplio300 [23]. Each image is provided with a detailed delineation of the nodule. For preprocessing, we crop the thyroid nodule area in ultrasound images and resize the areas to 256×256 in the dimension. The dataset was randomly split into a training set (5830 images), a verification set (729 images) and a test set (729 images) in a ratio of 8:1:1.

#### 3.1.2. TN3k

In order to facilitate the development of thyroid nodule segmentation, Gong et al. [5] contributed an open access dataset of thyroid nodule images called TN3k. This dataset includes 3493 ultrasound images taken from 2421 patients, and each patient retains only one ultrasound image of a case. Each image is converted into grayscale and corresponds to a high-resolution mask labeling. Compared with the TN-SCUI2020 dataset, this dataset has fewer data and lower ultrasound image quality. All images are randomly cropped to 256×256 before entering the models. To verify the performance of the network, we divided the TN3k dataset into the training set (2879 images) and test set (614 images).

### 3.2. Evaluation Metrics

Different metrics are employed to test the results of the proposed model. The performance of the thyroid nodule segmentation will be evaluated by dice, Jaccard, recall and precision, which are defined as:

(12) $$\mathrm{Dice}\left(\%\right)=\frac{2\mathrm{TP}}{2\mathrm{TP}+\mathrm{FP}+\mathrm{FN}}\times 100$$

(13) $$\mathrm{PA}\left(\%\right)=\frac{\mathrm{TP}+\mathrm{TN}}{\mathrm{TP}+\mathrm{TN}+\mathrm{FP}+\mathrm{FN}}\times 100$$

(14) $$\mathrm{Jaccard}\left(\%\right)=\frac{\mathrm{TP}}{\mathrm{TP}+\mathrm{FP}+\mathrm{FN}}\times 100$$

(15) $$\mathrm{Recall}=\frac{\mathrm{TP}}{\mathrm{TP}+\mathrm{FN}}$$

(16) $$\mathrm{Precision}=\frac{\mathrm{TP}}{\mathrm{TP}+\mathrm{FP}}$$

(17) $$\mathrm{FPR}=\frac{\mathrm{FP}}{\mathrm{FP}+\mathrm{TN}}$$

(18) $$\mathrm{FNR}=\frac{\mathrm{FN}}{\mathrm{TP}+\mathrm{FN}}$$

where TP, FP, FN and TN, respectively, represent the true positive, false positive, false negative and true negative, which are commonly used in image processing. Dice is also called the dice similarity coefficient, which can be used to compare the similarity between ground truth and prediction [24]. The PA is called pixel accuracy, which represents the proportion of pixels with the correct prediction class out of the total number of pixels. Jaccard, also known as intersection over union, is the primary measure of overlap between ground truth and segmentation results [25]. Furthermore, since our designed thyroid nodule segmentation network needs to assist radiologists in clinically diagnosing the disease, recall and precision are also included in our comprehensive experiments. Moreover, false positive rates (FPR) and false negative rates (FNR) are also the most commonly used evaluation metrics of thyroid nodule segmentation model, which are of great significance for practical clinical practice.

### 3.3. Implementation Details

The hyperparameters of each experiment in this work are generally consistent. During training, these experiments appropriately use data enhancement and apply data enhancement with a probability of 10/11. The used transformations are in rotation with an angle in degrees θ∼U(−10,10), horizontal flip with a possibility of 0.5. For our self-attention mechanism, we set the values of s to 1. The AdaBound [26] optimizer is employed, along with the Cosine annealing learning rate scheduler with a batch size of 16 and an initial learning rate of $1\times 10^{-3}$ until convergence. We train all models from scratch for 200 epochs on four GeForce RTX 2080Ti.

### 3.4. Comparison to State-of-the-Art Models

In this section, we compare our proposed LCA-Net with other state-of-the-art thyroid nodule segmentation networks, such as SGU-Net [4], TRFE-Net [6], RUL-Net [5] and WU-Net [27]. The SGU-Net based on UNet extracts single-channel pixel semantic maps from the high-dimensional features of each decoding step, which serves as a high-level semantic guidance to low-level features for capturing more details. The TRFE-Net simultaneously predicts the segmentation of thyroid regions and nodules, forcing the same backbone network to infer the localization of nodules. The RUL-Net integrates a modified UNet with a level set evolution as the segmentation network. The WU-Net uses the DenseNet-121 network as the backbone network and integrates the Atrous spatial pyramid pooling (ASPP) module for thyroid nodule segmentation in ultrasound images. Experiments are conducted under the same loss function, optimizer and GPU conditions to ensure fairness.

**Figure 7:**
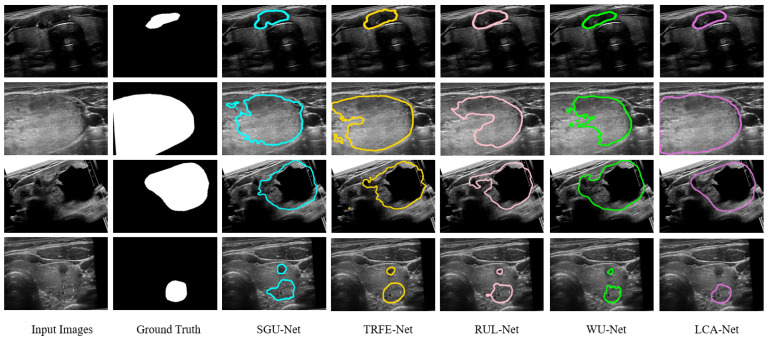
Qualitative comparison of different models on the TN-SCUI2020 dataset. Our predictions are closest to the ground truth and keep finer information.

**Figure 8:**
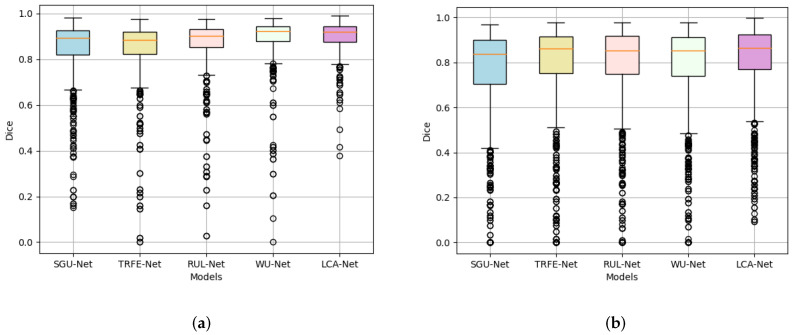
Boxplot of dice scores for each segmentation model. (**a**) Boxplot of dice scores on the TN-SCUI2020 dataset; and (**b**) boxplot of dice scores on the TN3k dataset. Median, interquartiles, minimum and maximum dice are provided.

Firstly, we calculate the nodule segmentation evaluation metrics defined above based on the TN-SCUI2020 dataset, and the comparison results for all methods are listed in Table 1. It can be seen from Table 1 that the proposed LCA-Net outperforms almost all state-of-the-art methods. Compared with several other models, the WU-Net has dramatically achieved the segmentation effect of thyroid nodules in ultrasound images (89.07% for dice, 98.24% for PA, 81.13% for Jaccard, 0.8957 for precision, 0.9082 for recall, 0.0076 for FPR and 0.1207 for FNR). However, thanks to the performance of LCA-Net for extracting both local details and global context information, the proposed LCA-Net achieves a certain improvement and reaches 2.48% for dice, 0.27% for PA, 3.06% for Jaccard, 0.0197 for precision, 0.027 for recall, 0.0013 for FPR and 0.0252 for FNR, respectively. The SGU-Net achieves poor results with 84.21%, 97.02%, 74.47%, 0.8346 and 0.8568 for dice, PA, Jaccard, precision and recall, respectively. The metrics of our method achieve a remarkable improvement and reach 90.26%, 98.87%, 82.65%, 0.9068 and 0.9184, respectively. Several examples of segmentation results with different methods are shown in Figure 7, from which it can be seen that our proposed method achieves the best thyroid nodule segmentation results. We select four different cases of thyroid nodules: a small nodule at the edge of the image, a large nodule at the edge of the image, a large nodule at the center of the image and a medium nodule at the center of the image. Other segmentation models have better results in the first row of the small nodule, except SGU-Net. For the nodule in the second row, the segmentation results of other models are far from the ground truth, while LCA-Net can still accurately segment the contour at the edge of the image and maintain good regional continuity owing to its ability to capture global associative information. Additionally, LCA-Net can still achieve better segmentation results than other models for the large nodule in the middle of the image. This is mainly due to the expansion of the receptive field of the backbone module for extracting detailed features and the flexible adaptive convolutions of the NAC module. For the medium-sized nodules in the last row, other models mistakenly segment two small nodules, which can easily cause major medical accidents in clinics. Fortunately, our model segments the more accurate nodule contour without misdiagnosis. In summary, the proposed LCA-Net has high segmentation accuracy and strong robustness to various nodules. Moreover, a boxplot diagram of the dice scores based on 5-fold cross verification has been presented in Figure 8a. Our model has the superiorities of median, interquartile and maximum dice values and has the least abnormal values, proving strong robustness for the segmentation of various nodules in the test set.

**Figure 9:**
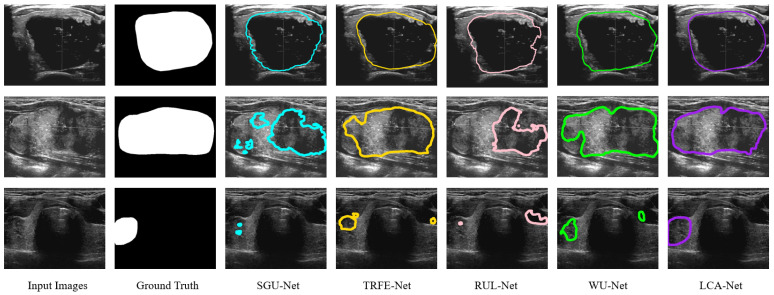
Qualitative comparison of different models on the TN3k dataset.

In addition, to further prove the generalization performance and effectiveness of the models, we conducted a new set of segmentation experiments on the TN3k dataset. The calculated evaluation metrics and experimental conditions are precisely the same as above. The specific experiment results are illustrated in Table 1. Although the TN3k dataset has a small amount of data and low image quality, the proposed LCA-Net achieves the best nodule segmentation results, including 82.08% for dice, 96.97% for PA, 71.18% for Jaccard, 0.8055 for precision, 0.8534 for recall, 0.0138 for FPR and 0.1473 for FNR. Other state-of-the-art models cannot perform very stably on this dataset compared with our outstanding results, proving that LCA-Net has good generalization performance for different datasets. Figure 9 also reflects the visualization results of each model nodule segmentation. We selected three examples: a medium-sized nodule in the middle of the image, a large nodule whose outline is all at the edge of the image, and a small nodule at the edge. It can be seen from the figure that our model can still more finely segment the thyroid contour. This also attributes the success to the capability of the context-attention modules to extract global features and the NAC module to adapt to nodules of various sizes and locations. Similarly, the boxplot diagram of the dice scores based on five-fold cross verification in Figure 8b also reflects the segmentation accuracy and generalization performance of our model. It can be seen from the figure that LCA-Net has minor abnormal points in the test results. Hence, it has good robustness for various types of nodule images in the test set. Simultaneously, these experiments also further demonstrate that our model has great application value in practical clinical thyroid segmentation.

Finally, we mixed the two datasets to further test the generalization ability and clinical application value of the model. We utilized the TN-SCUI2020 dataset as the training set and the TN3k dataset as the test set for experiments and vice versa. The specific experimental results are shown in Table 2. We can find that the proposed LCA-Net still achieves the highest test segmentation accuracy when the training set is completely different from the test set. This also proves that the LCA-Net has a strong application value and development prospects in clinical medicine.

### 3.5. Ablation Studies

In this section, in order to thoroughly evaluate the proposed LCA-Net framework and verify the performance of the selected loss function and optimization method, a variety of ablation studies were carried out on the TN-SCUI2020 dataset. Considering the fact that the TN-SCUI2020 dataset has wider data sources and more clinical representation than the TN3k dataset, we only use TN-SCUI2020 as the dataset for ablation experiments.

#### 3.5.1. Effect of Modules Selection

Since our proposed LCA-Net integrates the merits of context-attention modules, backbone modules and a nodule adaptive convolutions module concurrently to design the model, it is essential to investigate the impact of each kind of module on the performance of LCA-Net. We conduct this ablation experiment and calculate the metrics with different modules selections. The results are summarized in Table 3. Compared to just using the backbone modules, combining backbone and context-attention modules achieves an improvement in the main evaluation metrics (2.83% for dice, 0.39% for PA, 3.06% for Jaccard, 0.0154 for precision, 0.0229 for recall, 0.0057 for FPR and 0.0219 for FNR), which benefit from the fact that the context-attention modules can capture more global context information to improve the segmentation performance for nodule contours at image boundaries. In addition, the adoption of the nodule adaptive convolutions module is the best means of enhancing the generalization of the model, which further gains breakthroughs in the evaluation metrics (1.04% for dice, 0.23% for PA, 1.76% for Jaccard, 0.0164 for precision, 0.0146 for recall, 0.0011 for FPR and 0.0173 for FNR). However, due to the characteristics of ultrasound images and the complexity of thyroid nodule structure, the lack of backbone modules in the network to extract local details easily leads to a declining segmentation performance. Moreover, it can also be seen from the above that these experiments indicate the effectiveness of the module’s design and the combination of the models.

**Figure 10:**
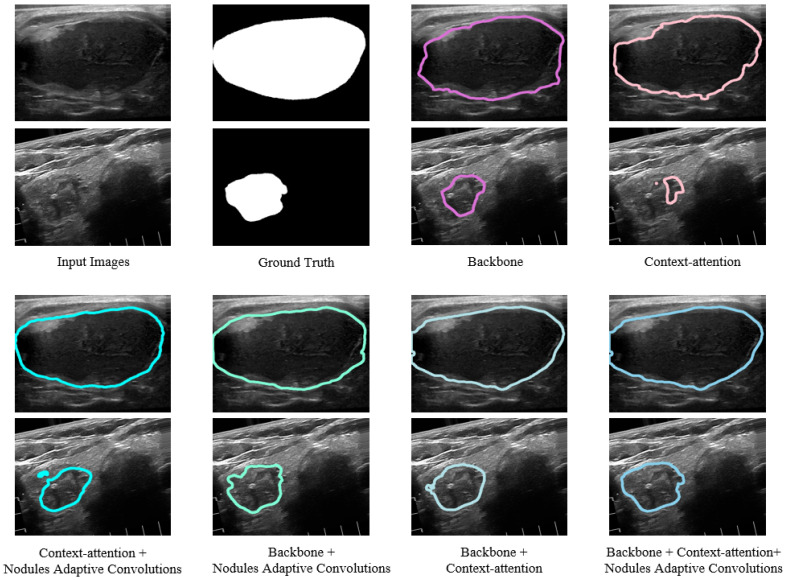
Qualitative comparison of various modules selections by visualization. A large nodule at the center of the image and a small nodule at the edge of the image are selected. The predictions of the complete network structure of LCA-Net are closest to the ground truth and keep more delicate information.

Several examples of segmentation results with modules variations are shown in Figure 10. Two different cases of thyroid nodules are selected: a large nodule at the center of the image and a small nodule at the edge of the image. If context-attention modules or context-attention modules and NAC module are selected, it is easy to cause the segmentation result of nodules imprecisely. Simultaneously, only employing the backbone modules or backbone modules and the NAC module will make the contour segmentation at the edge of the image less effective because the network cannot capture global context features. However, combining the context-attention modules and the backbone modules makes it possible to segment both edge contours and local details better. Furthermore, incorporating the NAC module further enhances the robustness of the network for the various nodules. It can be seen from these images that the proposed complete network structure can segment the nodule contour more finely and present more details, which effectively improves the accuracy of thyroid nodule segmentation and reduces false positives. In conclusion, our network structure is highly reasonable and has solid clinical value.

#### 3.5.2. Effect of Loss Function

Furthermore, we demonstrate the effect of different loss functions on the segmentation performance of the model. In most ultrasound images, the background size is more significant than that of the nodule, which can easily lead to the problem of class imbalance during segmentation and make it difficult to continue training. The classical dice loss [28] is often employed for medical image segmentation, but its performance in small target segmentation is not ideal. Once the small target has some pixel prediction errors, it will significantly change the loss function, resulting in a drastic change in the gradient and the instability of training. Meanwhile, due to the same weight assigned by the class, the binary cross-entropy (BCE) loss function cannot adequately deal with the imbalance of pixel class.

**Figure 11:**
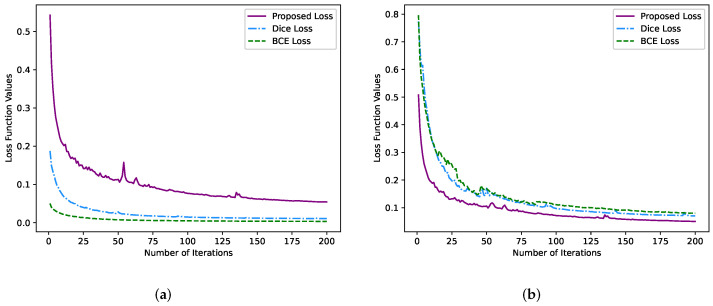
The various loss function curves with the number of iterations. (**a**) Loss function in training stage; and (**b**) Loss function in validation stage.

This paper compares the segmentation results of binary cross-entropy loss, dice loss and our proposed loss function under the same network structure and AdaBound optimizer. It can be seen from Table 4 that the evaluation metrics (such as dice, PA and Jaccard) of our proposed loss function are higher than the segmentation results of the dice loss function and binary cross-entropy loss function. It is worth noting that there is a significant gap in the precision and recall of the test results of the models obtained by the dice and BCE loss function. The main reason is that neither solves the problem of pixel class imbalance. Moreover, the segmentation of thyroid nodules in clinics has high requirements for both precision and recall. The variation trend of each loss function is shown in Figure 11a,b. By comparing the two figures, we can infer that the BCE loss and dice loss functions make the model over-fitting in the training stage. Although the proposed loss function curve has a few fluctuations in the middle process, it is generally stable. Meanwhile, the loss function curve converges faster, and the final function value remains in a reasonable range. In summary, the overall performance of our proposed loss function is the best. Therefore, our novel loss function is suitable for optimizing the thyroid nodule segmentation model.

#### 3.5.3. Effect of Optimizer Selection

Finally, we also conducted a set of ablation experiments for optimizer selection. The optimizer method is employed to assist in training to obtain the best network parameters and find the minimum loss of the loss function. Extensive experiments have shown that the SGD [29] optimizer has a slow convergence speed during training, which causes the result to easily fall into a local minimum. Simultaneously, the generalization of the model trained by the Adam [30] optimizer is not ideal. The AdaBound optimizer combines the merits of the SGD optimizer and Adam optimizer. At the beginning of training, it can be as fast as Adam and has as good a convergence as SGD in the later stage. This paper uses the SGD optimizer, Adam optimizer and AdaBound optimizer to train the LCA-Net under our proposed loss function. We select the appropriate optimizer by comparing different test results. It can be seen from Table 5 that the AdaBound optimizer achieved the best results in the evaluation indexes, which is higher than the commonly used SGD optimizer (3.49% for dice, 0.61% for PA, 3.6% for Jaccard, 0.0312 for precision and 0.0362 for recall). In addition, the test results using the Adam optimizer are not ideal, which proves that Adam is not suitable as the optimizer of the proposed model.

**Figure 12:**
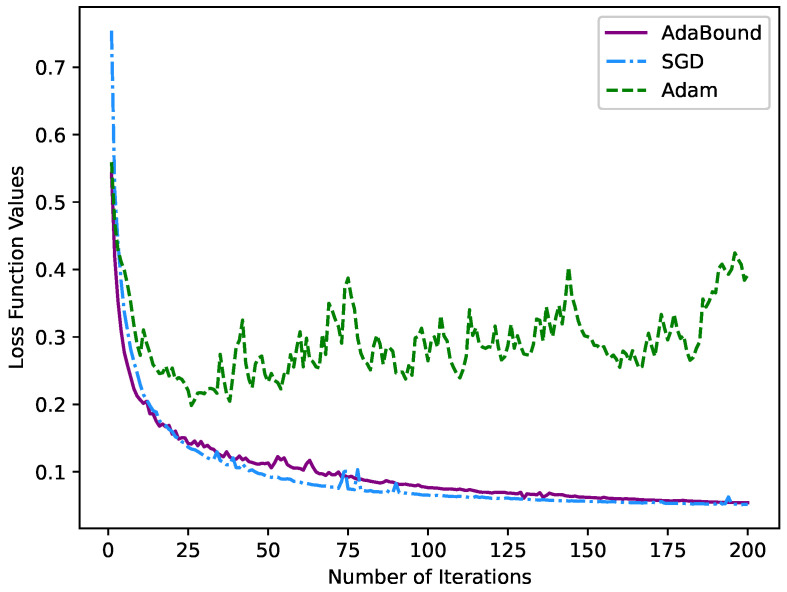
The novel loss function curve under different optimizers with the number of iterations.

The optimization of our proposed loss function by the three optimizers is shown in Figure 12. Compared with the SGD optimizer, the loss function curve corresponding to the AdaBound optimizer drops rapidly in the initial stage. As the number of iterations increases, the loss function tends to converge smoothly in the later stage. Meanwhile, the fluctuation of the loss function corresponding to the Adam optimizer is considerable, proving that the Adam optimizer is not suitable for the optimization of our model. In conclusion, our choice of the AdaBound optimizer for training the model is sensible and superior.

## 4. Conclusions

In this paper, a local and context-attention adaptive network (LCA-Net) for thyroid nodule segmentation in ultrasound images is proposed. The transformers-based context-attention module is proposed to capture more global associative information and perceive edge information of nodule contours to make up for the deficiency of convolutional neural networks in capturing context information. Additionally, the backbone module enables the network to extract more local features. Furthermore, a nodule adaptive convolutions (NAC) module is designed to deal with thyroid nodules of different sizes and positions adaptively. For the training of the model, an optimized loss function for pixel class imbalance is introduced to further enhance the segmentation performance. Compared with the latest algorithms, our method achieves promising segmentation accuracy and strong generalization performance. We believe that the proposed model could also serve other medical image segmentation tasks and has broad application prospects for diagnosing thyroid nodules.

## Figures and Tables

**Table 1:** Comparison of the results of state-of-the-art thyroid nodule segmentation models on the TN-SCUI2020 dataset and TN3k dataset.

Dataset	Model	Dice	PA	Jaccard	Precision	Recall	FPR	FNR
TN-SCUI2020	SGUNet [3]	84.21%	97.02%	74.47%	0.8346	0.8568	0.0111	0.1328
	TRFE-Net [5]	85.03%	98.04%	75.48%	0.8639	0.8652	0.0093	0.2252
	RUL-Net [4]	86.59%	97.97%	78.07%	0.8760	0.8812	0.0082	0.1543
	WU-Net [27]	89.07%	98.24%	81.13%	0.8957	0.9082	0.0076	0.1207
	**Proposed Method**	**90.26%**	**98.87%**	**82.65%**	**0.9068**	**0.9184**	**0.0063**	**0.0955**
TN3k	SGUNet [3]	75.03%	95.35%	64.25%	0.7719	0.7936	0.0158	0.2149
	TRFE-Net [5]	77.56%	96.23%	67.68%	0.7838	0.8316	0.0143	0.1915
	RUL-Net [4]	78.08%	96.72%	68.05%	0.8024	0.8113	0.0175	0.1606
	WU-Net [27]	81.27%	96.82%	70.54%	0.7822	0.8395	0.0195	0.1843
	**Proposed Method**	**82.08%**	**96.97%**	**71.18%**	**0.8055**	**0.8534**	**0.0138**	**0.1473**

**Table 2:** Comparison of the results of state-of-the-art thyroid nodule segmentation models of generalization performance.

Train Dataset	Test Dataset	Model	Dice	PA	Jaccard	Precision	Recall	FPR	FNR
TN-SCUI2020	TN3k	SGUNet [3]	81.37%	95.24%	70.06%	0.8077	0.8141	0.0167	0.2168
		TRFE-Net [5]	83.24%	96.49%	72.36%	0.8292	0.8201	0.0146	0.2058
		RUL-Net [4]	85.21%	97.08%	75.55%	0.8537	0.8574	0.0113	0.1599
		WU-Net [27]	87.78%	98.11%	78.32%	0.8746	0.8603	0.0117	0.1341
		**Proposed Method**	**89.82%**	**98.77%**	**80.97%**	**0.8913**	**0.8986**	**0.0085**	**0.1039**
TN3k	TN-SCUI2020	SGUNet [3]	71.03%	94.66%	62.27%	0.7478	0.7537	0.0219	0.2836
		TRFE-Net [5]	75.22%	95.93%	65.09%	0.7845	0.7813	0.0163	0.2917
		RUL-Net [4]	75.17%	95.77%	65.95%	0.7773	0.7682	0.0205	0.1829
		WU-Net [27]	77.32%	95.41%	68.21%	0.7914	0.8065	0.0218	0.2274
		**Proposed Method**	**78.86%**	**96.60%**	**69.43%**	**0.8128**	**0.8126**	**0.0145**	**0.1621**

**Table 3:** Comparison of thyroid nodule segmentation performance using various modules combinations.

Dataset	Module Construction			Results						
	Backbone	Context-Attention	Nodule Adaptive	Dice	PA	Jaccard	Precision	Recall	FPR	FNR
TN-SCUI2020	✓			86.39%	98.25%	77.83%	0.8750	0.8809	0.0131	0.1347
		✓		84.53%	97.65%	75.51%	0.8542	0.8320	0.0138	0.1435
		✓	✓	85.81%	97.94%	76.19%	0.8629	0.8695	0.0109	0.1253
	✓		✓	87.11%	98.44%	78.92%	0.8838	0.8897	0.0082	0.1266
	✓	✓		89.22%	98.64%	80.89%	0.8904	0.9038	0.0074	0.1128
	✓	✓	✓	**90.26%**	**98.87%**	**82.65%**	**0.9068**	**0.9184**	**0.0063**	**0.0955**

**Table 4:** Comparison of thyroid nodule segmentation performance using various loss functions.

Dataset	Loss Function	Dice	PA	Jaccard	Precision	Recall	FPR	FNR
TN-SCUI2020	BCE Loss	83.55%	98.31%	74.20%	0.6035	0.9672	0.0083	0.1496
	Dice Loss [28]	86.17%	98.49%	77.41%	0.7419	0.9539	0.0065	0.1321
	**Proposed Loss**	**90.26%**	**98.87%**	**82.65%**	**0.9068**	**0.9184**	**0.0063**	**0.0955**

**Table 5:** Comparison of thyroid nodule segmentation performance using various optimizers.

Dataset	Optimizer	Dice	PA	Jaccard	Precision	Recall	FPR	FNR
TN-SCUI2020	Adam [30]	78.93%	96.93%	68.41%	0.7901	0.8594	0.0213	0.1434
	SGD [29]	86.77%	98.26%	79.05%	0.8756	0.8822	0.0075	0.1215
	**AdaBound** [26]	**90.26%**	**98.87%**	**82.65%**	**0.9068**	**0.9184**	**0.0063**	**0.0955**
